# Efficacy of RNA polymerase II inhibitors in targeting dormant leukaemia cells

**DOI:** 10.1186/2050-6511-14-32

**Published:** 2013-06-15

**Authors:** Monica Pallis, Francis Burrows, Abigail Whittall, Nicholas Boddy, Claire Seedhouse, Nigel Russell

**Affiliations:** 1 Nottingham University Hospitals, Nottingham, UK; 2 Tragara Pharmaceuticals, San Diego, USA; 3 University of Nottingham, Nottingham, UK; 4 Academic Haematology, Clinical Sciences Building, Nottingham University Hospitals City Campus, Nottingham NG5 1PB, UK

**Keywords:** Leukemia, Dormancy, RNA polymerase II inhibitors

## Abstract

**Background:**

Dormant cells are characterised by low RNA synthesis. In contrast, cancer cells can be addicted to high RNA synthesis, including synthesis of survival molecules. We hypothesised that dormant cancer cells, already low in RNA, might be sensitive to apoptosis induced by RNA Polymerase II (RP2) inhibitors that further reduce RNA synthesis.

**Methods:**

We cultured leukaemia cells continuously *in vitro* in the presence of an mTOR inhibitor to model dormancy. Apoptosis, damage, RNA content and reducing capacity were evaluated. We treated dormancy-enriched cells for 48 hours with the nucleoside analogues ara-C, 5-azacytidine and clofarabine, the topoisomerase targeting agents daunorubicin, etoposide and irinotecan and three multikinase inhibitors with activity against RP2 - flavopiridol, roscovitine and TG02, and we measured growth inhibition and apoptosis. We describe use of the parameter 2 × IC_50_ to measure residual cell targeting. RNA synthesis was measured with 5-ethynyl uridine. Drug-induced apoptosis was measured flow cytometrically in primary cells from patients with acute myeloid leukaemia using a CD34/CD71/annexinV gating strategy to identify dormant apoptotic cells.

**Results:**

Culture of the KG1a cell line continuously in the presence of an mTOR inhibitor induced features of dormancy including low RNA content, low metabolism and low basal ROS formation in the absence of a DNA damage response or apoptosis. All agents were more effective against the unmanipulated than the dormancy-enriched cells, emphasising the chemoresistant nature of dormant cells. However, the percentage of cell reduction by RP2 inhibitors at 2 × IC_50_ was significantly greater than that of other agents. RP2 inhibitors strongly inhibited RNA synthesis compared with other drugs. We also showed that RP2 inhibitors induce apoptosis in proliferating and dormancy-enriched KG1a cells and in the CD71^neg^ CD34^pos^ subset of primary acute myeloid leukaemia cells.

**Conclusion:**

We suggest that RP2 inhibitors may be a useful class of agent for targeting dormant leukaemia cells.

## Background

Relapse in cancer patients after therapy is due to the continued presence of a subset of cells which is likely to have evaded the effects of treatment by lying dormant [1,2]. Dormant cells are characterised by low levels of RNA, consistent with their lack of proliferation and need to conserve energy [3]. However, cancer cells may be dependent on (“addicted to”) survival gene expression [4,5] and thus be primed for death if the survival genes are down-regulated [6]. Hence we hypothesised that dormant cancer cells, in which RNA levels are already low, may be sensitive to agents that target the transcriptional machinery. Transcriptional cyclin dependent kinases, i.e. CDK9 and CDK7, are permissive for transcription through modulation of the essential RNA elongation factor RNA Polymerase II (RP2). RP2 serine 5 phosphorylation by CDK7 normally occurs early in the initiation of transcription, whereas RP2 serine 2 phosphorylation by CDK9 predominates later, during elongation and termination [7]. Inhibition of RP2, although ultimately fatal to all cells, can allow for a therapeutic window by selectively affecting molecules essential to cancer cell survival. Foremost candidates for this role are those molecules with a short message and protein half-life [4,8]. An emerging group of multi-kinase inhibitors such as flavopiridol, roscovitine and TG02 inhibit transcriptional CDKs and thus RP2 activation in cells from patients with haematological malignancies [9-15].

A large amount of material is needed to study mechanisms of drug targeting and resistance, but dormant cancer cells are rare. Thus *in vitro* models of the dormant subpopulation would be valuable. In contrast to primary samples, leukaemia cell lines are plentiful and highly proliferative, so we sought a suitable method of inducing dormancy in these cells.

MTOR is a critical mediator of cell cycle progression [16,17]. In normal cells, mTOR integrates nutrient and growth factor signals such that factor deprivation inhibits mTOR, allowing the cell to conserve resources, quiesce and survive. This paper first addresses the chemosensitivity of the KG1a cell line, which retains long-term viability and is undamaged by mTOR inhibition. We show that these cells, which have a CD34^+^CD38^-^, p-glycoprotein^+^ phenotype characteristic of leukaemic progenitor cells [18], are enriched for features of dormancy by mTOR inactivation. We treat unmanipulated and dormancy-enriched cells with the nucleoside analogues ara-C, 5-azacytidine and clofarabine, the topoisomerase targeting agents daunorubicin, etoposide and irinotecan and three multikinase inhibitors with activity against RP2 - flavopiridol, roscovitine and TG02. We report our findings and extend them to primary leukaemia samples.

## Methods

### Materials

Phenotyping antibodies and isotype controls were obtained from BD Biosciences. TG02-citrate was synthesised by Tragara Pharmaceuticals. Other drugs and reagents were obtained from Sigma unless otherwise stated.

### Cells and rapamycin pre-treatment

The KG1a myeloid leukaemia cell line was obtained from the European Collection of Animal Cell Cultures (Salisbury, UK) and was maintained in RPMI 1640 medium with 10% foetal calf serum (FCS; First Link, Birmingham, UK) and 2 mM L-glutamine. All experiments were performed with cell lines in log phase. Continued testing to authenticate the cells was performed by genetic fingerprinting towards the final passage of each batch thawed and through repeated assays of CD34, CD38 and p-glycoprotein status. The cells were pre-treated with rapamycin (LC labs) for 2–9 days before addition of chemotherapy drugs.

### Ethics statement

Blood or bone marrow samples were obtained after written informed consent from AML patients. Use of these samples was approved by the Nottingham 1 Ethics Committee (reference 06/Q2403/16) and the Nottingham University Hospitals NHS Trust. Frozen, banked samples were used.

### Drug treatment in cell lines

Unmanipulated and rapamycin-pre-treated KG1a cells were pelleted and re-suspended in 96 well plates at 2 × 10^5^ cells per ml for 48 hours with and without drugs. Cytosine arabinoside (Ara-C), flavopiridol, irinotecan and daunorubicin stock solutions were made in water. Clofarabine stock was made in PBS. 5-azacytidine, etoposide, roscovitine (LC labs) and TG02 were dissolved in DMSO as was the RP2 inhibitor 5,6-dicholoro-1-β-D-ribofuranoslybenzimidazole (DRB). DMSO diluent controls were used for etoposide and roscovitine (because the final DMSO concentration was greater than 1 in 10,000). Drug dilutions were made in culture medium.

### Determination of RNA status and RNA synthesis

For flow cytometry, the method of Schmid was used using 7-amino actinomycin D (7-AAD) to label DNA and pyronin Y to label RNA [19]. RNA was also measured on unselected cells by spectrophotometry. RNA synthesis was measured flow cytometrically using the method of Jao and Salic [20]: 5-ethynyl uridine (EU, Invitrogen) incorporation (20 μM, 1 hour) was followed by detection with Alexa 488 azide (Invitrogen). A non-specific fluorescence control tube, missing out the EU incorporation step, was set up for each condition, and the result subtracted from the test fluorescence value before calculating the percentage of untreated control fluorescence for each drug.

To determine modulation of RP2S2, treated and untreated cells were fixed and permeabilized using the Leucoperm kit (AbD Serotec) and were incubated with antibodies to RP2S2 (Abcam #5095,) then with a FITC conjugated second layer.

### Determination of reactive oxygen species (ROS)

Cells were incubated with the (non-fluorescent) 15 μM 2′,7′-Dichlorofluorescin diacetate (DCFDA) in triplicate for 25 minutes at 37°C and at 4°C, placed on ice and the fluorescent oxidation product dichlorofluorescin (DCF) was measured immediately by flow cytometry. Baseline (4°C) values were subtracted from test (37°C) values.

### Determination of metabolism

Cellular metabolism was measured using the reduction of 2,3-bis(2-methoxy-4-nitro5-sulfophenyl)-5-{(phenylamino)carbonyl}-2*H*-tetrazolium hydroxide (XTT, Roche) [21]. Cells were plated at 2 × 10^5^/ml and cultured for 48 hours, with XTT for the final 6 hours. Relative absorbance was calculated after adjustment for final cell concentration (measured by haemocytometer).

### Immunocytochemistry

Gamma-H2A.X foci were identified and counted using the H score system as previously described [22].

### Determination of cell viability and apoptosis in cell lines

Toxicity was measured using the XTT assay kit according to manufacturer’s instructions (Roche). Apoptosis was measured flow cytometrically using the Trevigen Annexin V kit (R & D) according to manufacturers’ instructions.

### Dormancy and apoptosis of primary AML cells

Primary cells were cultured in triplicate at 1 × 10^6^/ml in fibronectin coated wells of a flat-bottomed plate in serum-free medium supplemented with cytokines. (A previous publication [23] has further details). Drugs were added after 2–3 hours. After 14–18 hours of further culture, cells were harvested and stained with CD34PerCP and CD45-APCCy7, and with CD71PE or isotype controls. Following two rinses in PBS, the cells were counterstained with Annexin V FITC in the buffer provided (R&D Systems). CD71 expression was measured in cells gated tightly on forward and side scatter, with secondary gating on CD45 and side scatter to exclude lymphocytes. A third gate was set on CD34/low side scatter and a fourth gate on annexin V low positive cells. To ensure at most a 15% co-efficient of variation, cultures with a low number of cells after this four-part gating, i.e. less than 50 positive events, were excluded (explained in detail elsewhere [24]).

### Statistical analysis

Univariate analysis of variance main effects modelling was used for comparing multiple treatments, and significant findings were further analysed in 2 way comparisons using paired T-tests, carried out using the Statistical Package for Social Sciences, version 16 (SPSS, Chicago, IL, USA).

## Results

### mTOR inhibition induces the principal features of dormant cells

**Figure 1:**
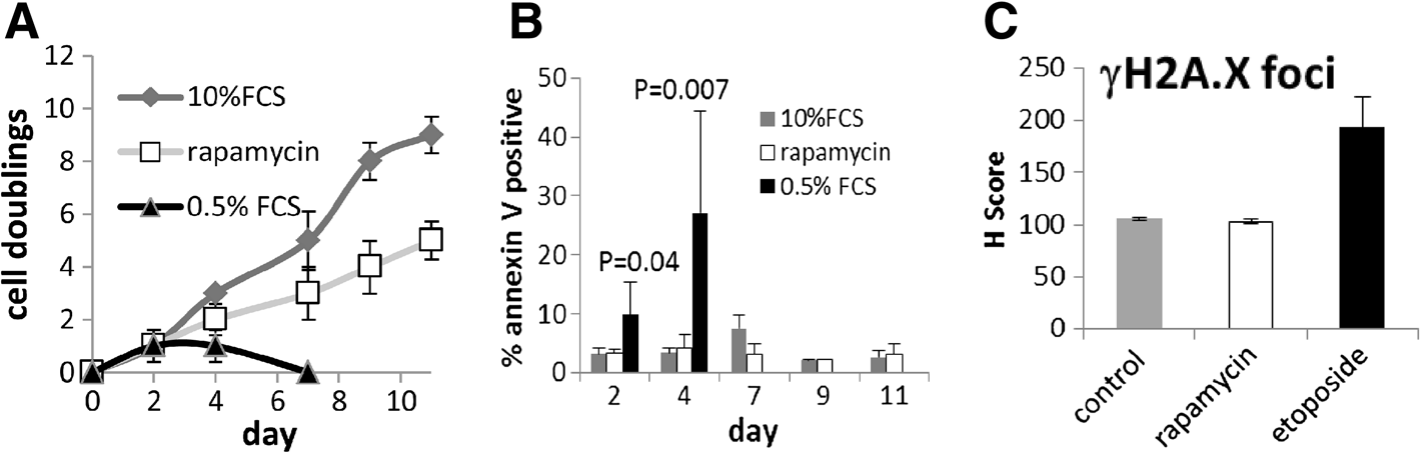
**Rapamycin is not toxic to KG1a cells.** (**A**, **B**) KG1a cells were suspended/re-suspended at 2 × 10^5^/ml on days 0,2,4,7 and 9 of an 11 day culture in medium with 0.5% foetal calf serum, 10% foetal calf serum or 10% foetal calf serum + 100 nM rapamycin. (**A**) Cell growth was measured by haemocytometer; (**B**) apoptosis was measured by Annexin V labelling. Annexin V was measured only at 2 and 4 days on cells grown in 0.5% FCS as there were insufficient cells remaining by 7 days. (**C**) Following 48 hours’ culture in RPMI with 10% foetal calf serum with or without 100 nM rapamycin, cells were harvested, and processed for immunohistochemical analysis of γH2A.X foci. Cells treated for 2 hrs with 100 μg/ml etoposide were used as positive control in each assay. (For interpretation, an H score (reference 22) is a staining intensity score of 1–5 per cell for 100 cells, such that a score of 100 represents no staining and a score of 500 represents 100 completely damaged cells.). In each experiment datapoints indicate mean and standard deviation of 3 independent assays.

Given that inhibition of the mTOR pathway is experimentally proven to maintain the *in vivo* dormancy and transplantability of haematopoietic and leukaemic cells [25-28], we experimented with the possibility of inhibiting growth in a leukaemic cell line with the mTOR inhibitor rapamycin. In preliminary studies, we cultured KG1a cells with 50-500 nM rapamycin and found similar percentage growth inhibition across the dose range (data not shown), such that 100 nM was chosen for further study. We now show that continuous culture of KG1a cells in 100 nM rapamycin for up to 11 days induced no detectable apoptosis, whereas serum withdrawal, the common method for inducing cells to exit the cell cycle, induced a statistically significant induction of Annexin V within 48 hours, and most cells were dead within a week (Figure 1A,B). Sublethal damage in the rapamycin-treated cells might sensitise them to chemotherapeutic drugs, but we determined that no measurable γH2A.X damage foci were induced by rapamycin (Figure 1C).

**Figure 2:**
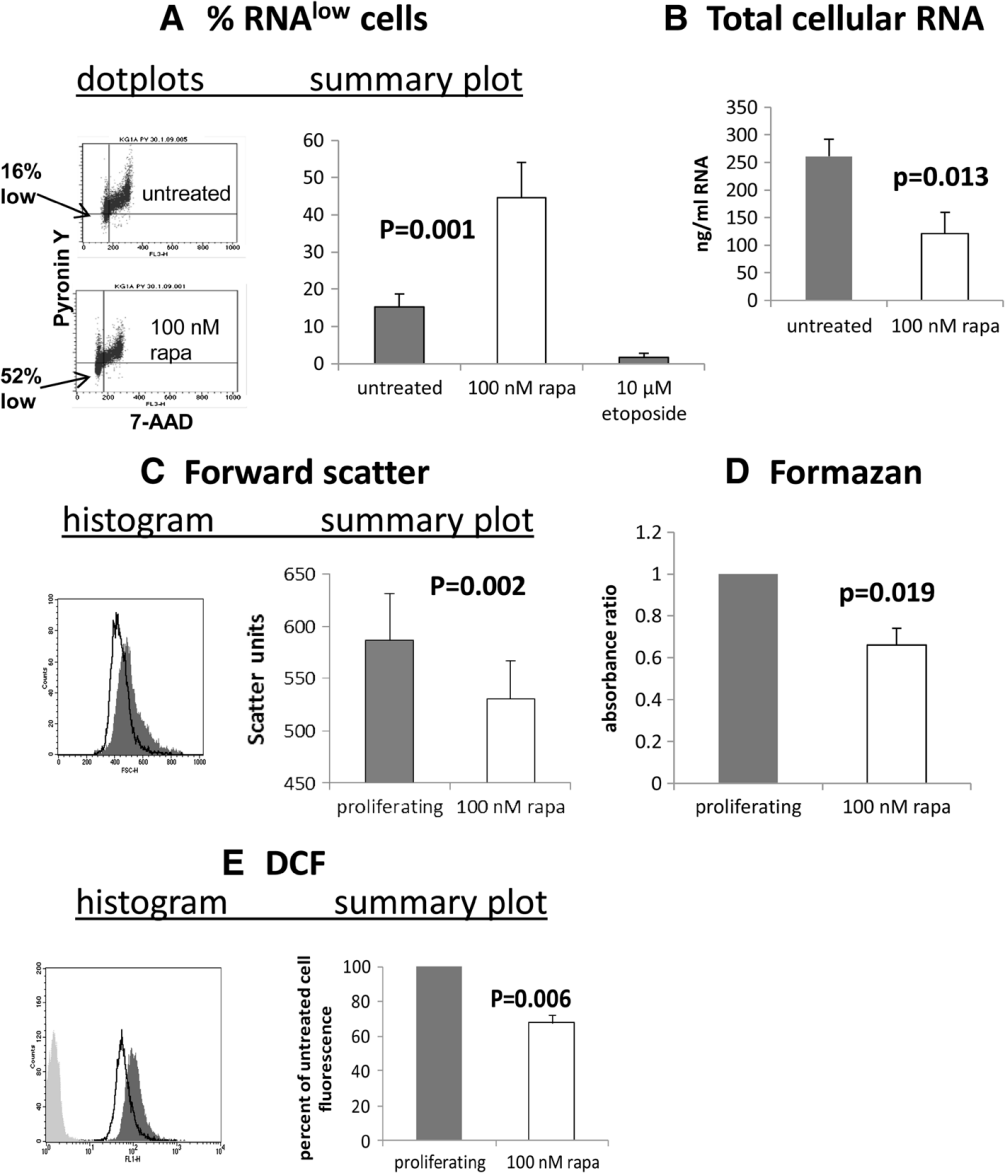
**Rapamycin induces key features of dormancy.** Evaluation of cellular properties after treatment of KG1a cells with 100 nM rapamycin for 48 hours. (**A**) RNA content: Flow cytometric dotplots indicate the percentage of cells which are low in RNA (Pyronin Y) as well as low in DNA (7-AAD) and summary chart. In the summary chart, the RNA content of KG1a cells treated for 48 hours with etoposide, which induces predominant G2/M arrest, are used as negative control. (**B**) The total RNA content of lysed cells, measured by spectrophotometry. (**C**) Flow cytometric analysis of forward scatter as an indicator of cell size: representative histogram: dark-filled histogram = untreated cells, unfilled histogram = rapamycin-treated cells and summary graph (instrument and voltage-dependent units). (**D**) Reduction of 2,3-bis(2-methoxy-4-nitro5-sulfophenyl)-5-{(phenylamino)carbonyl}-2*H*-tetrazolium hydroxide to formazan: absorbance ratio (adjusted for cell count) after 48 hours as a measure of mitochondrial metabolism. (**E**) Flow cytometric dichlorofluorescein diacetate as a measure of reactive oxygen species: example, dark-filled histogram = untreated cells, unfilled histogram = rapamycin-treated cells, pale grey histogram = baseline; summary graph. In each experiment datapoints illustrate mean and standard deviation of at least 3 independent assays. All P values are for comparisons between untreated and rapamycin-treated cells.

We have already previously shown that rapamycin inhibits phosphorylation of the mTOR targets 4E-BP1 and P70S6K in KG1a cells [29]. In a series of experiments performed after 48 hours’ incubation with rapamycin we found that, despite cell growth being slowed rather than totally arrested by rapamycin, the cells acquired key properties of dormant cells. There was a decrease in RNA, measured as a 3.5fold increase in Pyronin Y^low^ cells, from 13.6 to 48.6% cells and a decrease in total RNA per cell of 54% (Figure 2A). This is an especially important finding, as Pyronin Y^low^ cells are enriched for dormancy rather than terminal differentiation as demonstrated by their engraftment capacity in both normal haematopoietic cells and tumour initiating cells [30,31]. We also observed a corresponding decrease in cell size (Figure 2C) [3]. We noted that formazan production from XTT, an indicator of mitochondrial metabolism, was reduced by 34% in dormancy- enriched cells (Figure 2D). We also noted a 32% ROS decrease in dormancy-enriched cells (Figure 2E).

### Superiority of transcriptional CDK/RP2 inhibitors in targeting dormancy-enriched cells

**Figure 3:**
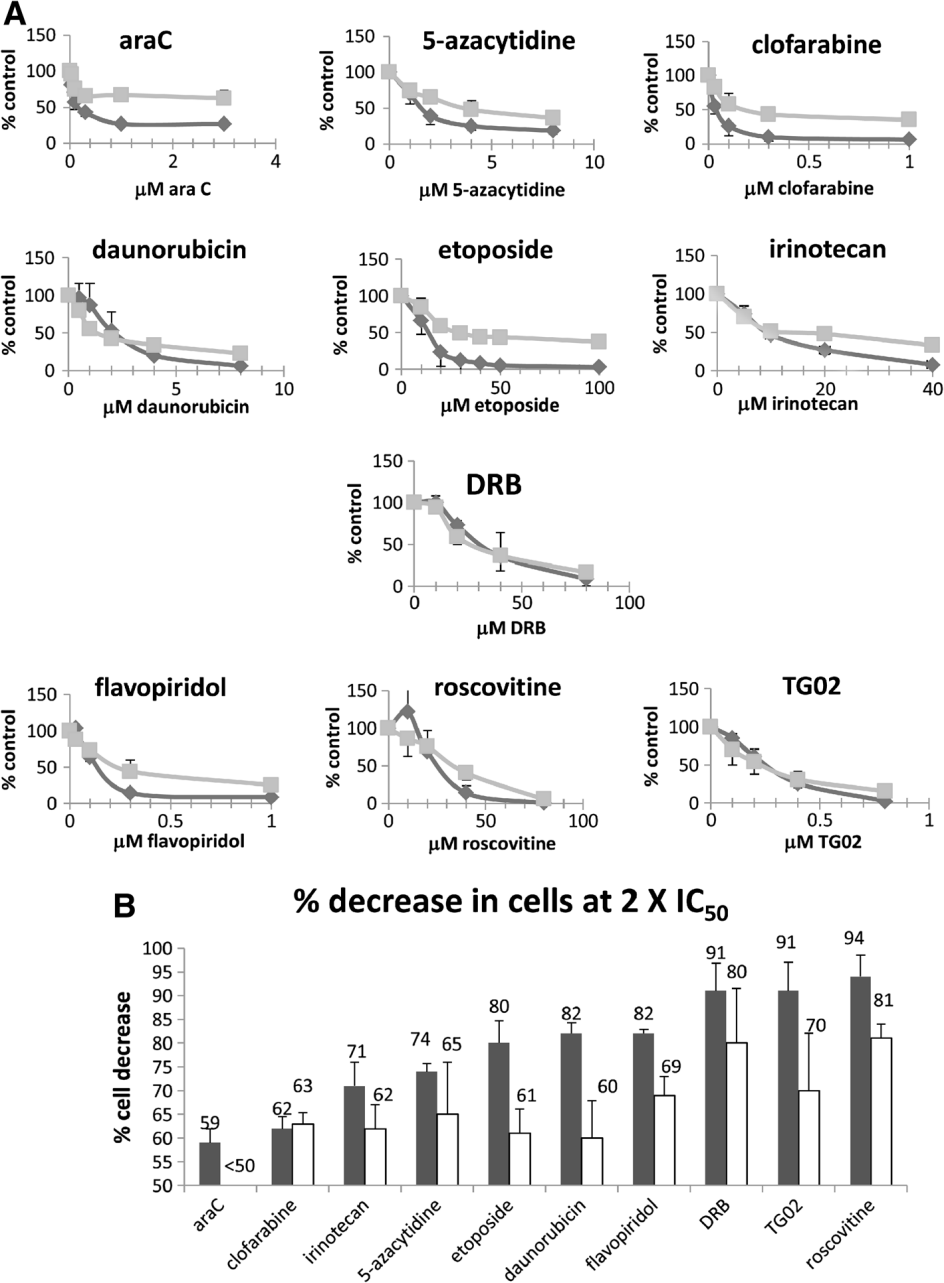
**KG1a responses to chemotherapeutic agents.** Un-manipulated KG1a cells and cells enriched for dormancy by rapamycin pre-treatment were cultured in the presence of drugs for 48 hours whereupon net cell growth and survival was estimated using the 2,3-bis(2-methoxy-4-nitro5-sulfophenyl)-5-{(phenylamino)carbonyl}-2*H*-tetrazolium hydroxide (XTT) assay. (**A**) Dose response curves in proliferating and dormancy-enriched cells. Dark lines represent un-manipulated cells and light grey lines represent dormancy-enriched cells. (**B**) Percentage decrease in viable cells at 2 × IC_50_. Dark bars represent unmanipulated cells and unfilled bars represent dormancy-enriched treated cells. Note that for araC no IC_50_ for dormancy-enriched cells was reached, even at 20 times the IC_50_ of proliferating cells. In each experiment datapoints indicate mean and standard deviation of at least 3 independent assays.

As nucleoside analogues and topoisomerase inhibitors are the mainstay of AML therapy, we examined the toxicity of these drug classes as well as that of RP2 inhibitors against unmanipulated and dormancy-enriched KG1a cells. We derived dose response curves for the topoisomerase- targeting agents daunorubicin, etoposide and irinotecan, nucleoside analogues ara-C, 5-azacytidine and clofarabine and the transcriptional CDK/RP2 inhibitors flavopiridol, roscovitine and TG02 in proliferating and dormancy-enriched KG1a cells. We also used the specific RNA polymerase 2 inhibitor 5,6-dicholoro-1-β-D-ribofuranoslybenzimidazole (DRB) as positive control for RP2 targeting [4,32]. Figure 3A demonstrates that dose response curves from dormancy-enriched cells have a greater tendency than those from unmanipulated cells to flatten out and there are more residual cells under the flattened part of the curve. We therefore asked a novel question: namely, how can we measure the difficulty for a drug to target further cells after the initial IC_50_ has been passed? For this measure, the parameter we used was cell reduction at 2 × IC_50_. Thus, using an example from Figure 3B, roscovitine reduces unmanipulated cell number by 94% at 2 × IC_50_, i.e. by doubling the IC_50_ concentration roscovitine managed to deplete a futher 44% of cells, whereas araC manages to deplete only a further 9% of cells when the IC_50_ concentration is doubled (Figure 3B). We established that the RP2 inhibitor group of drugs were significantly more effective at reducing cell number at 2 × IC_50_ than the topoisomerase targeting agents or the nucleoside analogues (P = 0.001 for topoisomerase targeting agents and P < 0.001 for nucleoside analogues compared with RP2 inhibitors in unmanipulated cells, P = 0.003 for both comparisons in dormancy-enriched cells, Figure 3B.) (DRB was used as a positive control for RP2 targeting and is not a chemotherapeutic agent: its effects were therefore not included in the statistical analysis).

### Targeting of RNA polymerase II and RNA sythesis by RP2 inhibitors

**Figure 4:**
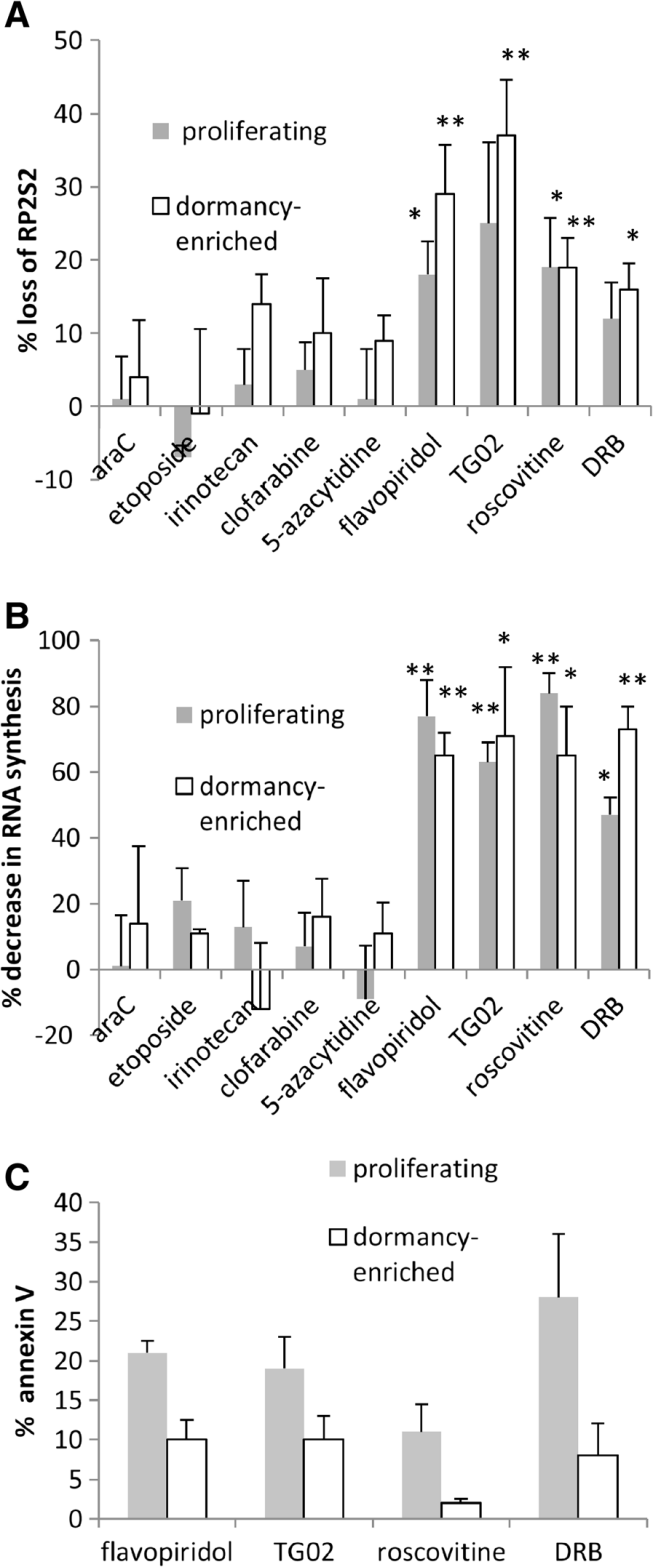
**Decreased RNA Polymerase II activity and RNA synthesis in transcriptional RP2i-treated KG1a cells.** Un-manipulated KG1a cells and cells enriched for dormancy by rapamycin pre-treatment were cultured with chemotherapy drugs, each drug at its 48 hour IC_50_ concentration. (Daunorubicin was not included in this set of experiments, due to interference from its fluorescent properties). (**A**) Loss of serine 2-phosphorylated RNA Polymerase II (RP2S2) was measured after 6 hours’ incubation of proliferating and dormancy-enriched cells with chemotherapeutic agents. For comparisons against untreated controls ** signifies P < 0.01; * signifies P < 0.05. (**B**) RNA synthesis was measured by 20 μM 5-ethynyl uridine incorporation for the final hour of a six hour treatment with chemotherapeutic agents. For comparisons against untreated controls ** signifies P < 0.01; * signifies P < 0.05. (**C**) Apoptosis was measured using Annexin V after 18 hours. In each experiment datapoints indicate mean and SEM of at least 3 independent assays.

Serine 2 of the elongation factor RNA Polymerase II (RP2S2) is a molecular target of CDK9 [33]. Flavopiridol, roscovitine and TG02 have multiple and diverse targets in addition to RP2. We therefore measured whether RP2S2 and RNA synthesis were being targeted at each drug’s IC_50_. The existing literature, including our own previous work with TG02 [10,13-15] indicated that investigation of these parameters after 6 hours of treatment would show optimal effects. At this timepoint, RP2S2 was significantly downregulated in dormancy-enriched KG1a cells treated with RP2 inhibitors (Figure 4A). RNA synthesis was greatly reduced at the same timepoint (Figure 4B). A number of molecules with short message and protein half-lives are depleted by RNA polymerase II inhibitors [8], including several survival and cycle-related proteins [11-13,15]. Moreover TG02, flavopiridol and roscovitine are all documented to induce cell cycle arrest in G0/G1 [14,34,35], which we confirmed in the KG1a model (data not shown), so it was important to establish whether the decreases in cell numbers relative to untreated controls were solely due to growth inhibition or whether, as would be necessary for dormant cell targeting, they also undergo apoptosis. We observed apoptosis in all cases at the IC_50_ for DRB, flavopiridol and TG02 (Figure 4C). Roscovitine had notably little ability to induce apoptosis in the dormancy-enriched cells (discussed below).

### Sensitivity to RP2 inhibition in dormant CD34+ primary leukaemic cells

**Figure 5:**
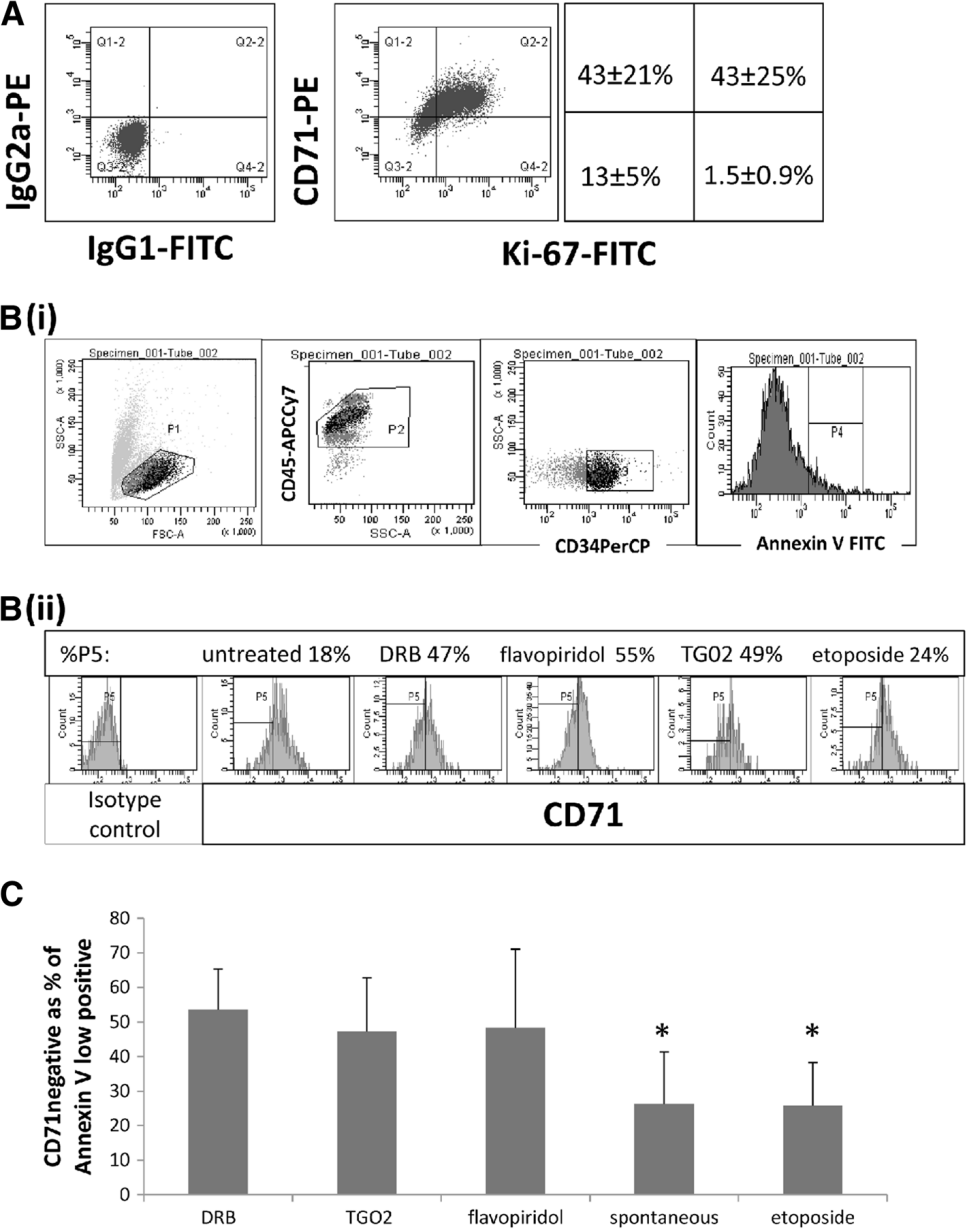
**Apoptosis in CD34+ dormant patient cells treated with RP2 inhibitors.** (**A**) Ki-67/CD71 co-expression in CD34-gated primary AML cells before culture. For Ki-67 and CD71 quadrant delineation, gating was carried out strictly such that 1% of isotype control fluorescence fell into the positive quadrants. Flow cytometric dotplots of one sample and a diagram summarising mean ± standard deviation of target cell percentage in each quadrant for the seven primary samples studied are shown. (**B**) An example of CD71 expression in CD34+ annexin V + AML blasts. Patient cells were cultured for 16–18 hours with DRB, TG02, flavopiridol or etoposide. They were then labelled with CD71, CD45 and CD34, rinsed and additionally labelled with Annexin V. CD71 expression was determined in CD34+ early apoptotic cells using the four part gating strategy detailed in the Methods section. (i) Illustration of gating strategy showing how gates P1-P4 are applied; note especially that gates P1 and P4 are narrowed to exclude late stages of apoptosis or necrosis in order to alleviate concern that CD71 might be shed. (ii) Flow cytometric histograms showing CD71 expression in the cell subset gated on P1-P4 (MFI = mean fluorescence intensity). (**C**) CD71 negative cells shown as a percentage of total early apoptotic cells from the P1-P4 subset of primary samples (n = 8 for TG02 and etoposide, n = 6 for DRB and flavopiridol). As primary samples are heterogeneous, apoptosis-inducing drug concentrations were sample-specific (30-100 nM for TG02 and flavopiridol, 0.2-2 μM for etoposide, 20 μM for DRB). * The low proportions of CD71neg cells in untreated and etoposide-treated samples compared to RP2 inhibitor-treated samples were statistically significant, as detailed in the text.

We and others have previously documented the *in vitro* toxicity of TG02 to bulk CD34 + CD38- primary AML cells and demonstrated effective cell reduction at 100 nM [14,15]. The CD34 + CD38- subset is enriched for dormant cells, but to address directly the question of whether RP2 inhibitors target dormant primary cells, we sought a flow cytometric assay that would combine a dormancy marker with an apoptotic marker. Annexin V is the standard, extremely sensitive, marker for apoptosis in non-adherent cells, but its use in permeabilised cells is problematic. Ki-67 is the standard marker for excluding, and thus identifying, dormant cells, but detects an intracellular antigen and thus requires cells to be fixed and permeabilised, which compromises Annexin V staining. We looked for a cell surface marker which would discriminate between dormant and cycling cells and could be used in conjunction with Annexin V to investigate apoptosis in dormant cells. The absence of CD71, the transferrin receptor, has been reported in dormant lymphocytes and in cancer stem cells [36,37]. In preliminary experiments, we established that cells which were negative for CD71 (i.e. found in the lower two quadrants of the dot plots in Figure 5A) were also almost all Ki-67-negative (i.e. there were very few cells in the lower right quadrants). CD71 negative cells can therefore be classified as dormant. To determine whether RP2 inhibition induces apoptosis in dormant primary AML blasts, we labelled *in vitro*-treated blasts for Annexin V and CD71. Figure 5B shows our gating strategy. Using eight primary samples, we found clear evidence of CD71^neg^ cells in the Annexin V ^lowpos^ subset of CD34+ AML blasts treated with DRB, TG02 or flavopiridol (Figure 5C). When compared with etoposide (which had not been effective in targeting dormancy-enriched KG1a cells as shown in Figure 3) a significantly higher proportion of apoptosing cells was found in the CD71^neg^ compartment after treatment with all three RP2 inhibitors (P < 0.001 for DRB and TG02, P = 0.007 for flavopiridol). It is important to understand here that we are not comparing the toxicity of different agents but are selecting cells in which apoptosis is occuring to determine in which compartment (dormant or non-dormant) it is occuring. We also took advantage of the fact that primary AML cells *in vitro* show some spontaneous apoptosis, compared to which all three RP2 inhibitors again were associated with a significantly greater proportion of apoptotic cells in the CD71^neg^ compartment (P < 0.001 for DRB, P = 0.003 for TG02 and P = 0.01 for flavopiridol). Roscovitine at doses up to 2 μM only reduced viable cell concentration in a minority of primary samples studied and we therefore have not documented results with this agent.

## Discussion

There is a paradox at the centre of chemoresistance research, in that most anti-neoplastic drugs have been designed to target proliferating cells as a surrogate for tumour cells, and therefore the highly chemoresistant dormant tumour cell does not fit into the mainstream chemotherapeutic paradigm. In the AML field, a vanguard of researchers has been investigating possible solutions to this problem for the last twenty years and more. From *in vitro* and animal model work, it is clear that non-proliferating AML cells are resistant to ara-C, and that chemosensitivity increases when cells are induced into cycle by growth factor exposure [38-40]. However over a dozen clinical trials reflecting a great deal of effort in applying this knowledge have yielded equivocal results ([41] and references therein) and fresh approaches are needed.

We reasoned that it would be useful to have *in vitro* models to contribute to the search for ways of targeting dormant leukaemia cells. Whilst there is a need for the creation of suitable *in vivo* models to test longer- term chemosensitivity in dormant leukaemia cells in an appropriate microenvironment, we suggest that the value of *in vitro* work is that it allows the comparison of a broad range of drugs and allows for investigations of their mechanisms of action, as is illustrated in the current work.

The overwhelming evidence that activation of the mTOR pathway pushes haematopoietic and leukaemic cells out of dormancy [25-28] led us to investigate this pathway. Moreover an elegant study published after the current work was completed showed that AML cells with an undifferentiated phenotype have prolonged *in vivo* survival when mTOR activity is knocked out, and that subsequent mTOR re-activation restores the leukaemogenic potential of these cells [42]. In our study, rapamycin slowed, but did not completely arrest growth in KG1a cells (Figure 1A). However, in contrast to serum withdrawal, which is the most commonly used model for dormancy, rapamycin did not cause apoptosis or DNA damage (Figure 1) - an essential consideration for a model in which the chemosensitivity of previously undamaged cells is to be assessed. We suggest that rapamycin provides a useful model for dormancy because key features, i.e. low RNA, low metabolism and low ROS, are enriched in the rapamycin-treated compared with the untreated cells (Figure 2). Low RNA is of paramount importance, since cells characterised by low RNA content retain the capacity to re-enter the cell cycle and act as progenitors *in vivo*[30,31]. The RNA^low^ characteristic of dormant cells is consistent with their lack of proliferation and low metabolism [3], but for dormant cancer cells this might be difficult to reconcile with addiction to survival gene expression, leading us to suggest that these cells may be sensitive to transcriptional RP2 inhibitors. A publication several years ago showed that flavopiridol targets non-cycling A549 cells [43]. Flavopiridol and roscovitine were initially designed to target cyclin dependent kinases that drive cell proliferation, and it was only subsequently that the effects were noted for both of these agents on down-regulating survival molecules and inhibition of transcription through inactivation of CDK9 [9,10,13,44]. TG02 has been characterised more recently and, like the two other agents, has multiple targets including cycling and transcriptional CDKs [14]. In cell-free assays, TG02 has a 3nM IC_50_ for CDK9 [14]. As all three agents have multiple targets, we also used the RP2-specific inhibitor DRB in our assays [4,32].

We have shown that transcriptional RP2 inhibitors are better able than conventional agents to target dormancy-enriched AML cells. We hypothesised that a therapeutic window might exist for dormant cancer cells because of the addiction of malignant cells to survival gene expression [4,5]. Results from clinical trials with roscovitine and flavopiridol [45] including a trial incorporating flavopiridol in combination chemotherapy of AML [46] have shown some efficacy at sub-toxic doses. TG02 at tolerated doses induced lasting complete remissions in an AML xenograft model [14] and at the time of writing, is in Phase 1 trials for refractory and relapsed leukaemias. We show that the specific transcriptional RP2 inhibitor DRB as well as TG02, roscovitine and flavopiridol down-regulate RNA Polymerase II activation and RNA synthesis in both unmanipulated and dormancy-enriched cells. We have not attempted to pick out specific targets of RP2 down-regulation, as these are multitudinous [8]. Functionally they tend to be genes involved in rapid cellular responses, such as apoptosis regulators, mitosis regulators and genes involved in signalling pathways such as several NFκB target genes [8]. We have shown that apoptosis is induced by the specific RP2 inhibitor DRB and by flavopiridol and TG02 (Figures 4 and 5). Roscovitine appears to work mainly by growth inhibition or a non-apoptotic mechanism of death. It is noteworthy in this respect that gene expression profiling of agents inhibiting transcriptional CDKs found that DRB and flavopiridol had similar broad activity, whereas roscovitine had a narrower range of activity [8]. Moreover, in our hands, micromolar concentrations of roscovitine were found to reduce viable cell concentration in only 5/12 leukaemia samples studied *in vitro* (data not shown) in contrast to a robust response to TG02 at 100 nM [15].

To further validate our results indicating that RP2 inhibitors target dormancy-enriched KG1a cells, we sought agreement for our findings in primary material. In contrast to cell lines, primary AML samples are enriched for cells in G_0_ of the cell cycle [39]. We examined the extent of apoptosis induced by RP2 inhibitors in dormant and proliferative compartments of primary cells. As Annexin V is a highly sensitive marker for early apoptosis in primary AML cells, we looked for a cell surface dormancy marker that could be used in conjunction with Annexin V to measure apoptosis in dormant compared to proliferating cells. CD71 (the transferrin receptor) is absent in un-stimulated peripheral blood lymphocytes, in some cancer stem cells and in long term culture-initiating cells from normal bone marrow [36,37,47]. Analysis of patient samples co-labelled with CD71 and Ki-67 indicated that CD71 is not expressed in dormant AML blasts (Figure 5). Co-labelling of cells with CD71 and Annexin V clearly indicated the contrast between the high proportion of CD71^neg^ apoptotic cells following treatment with DRB, flavopiridol or TG02 and the high CD71 expression in etoposide-treated apoptotic cells. Even compensating for the plasma protein binding of the drug, the concentration of TG02 used in this experiment is readily achievable *in vivo* in both animals [14] and humans (as measured following oral administration in ongoing clinical studies - FB, unpublished).

## Conclusion

In conclusion, we have shown that RP2 inhibitors effectively target both KG1a cells enriched for dormancy by mTOR inhibition and CD71^neg^ primary leukaemia patient samples thus providing grounds for suggesting that transcriptional RP2 inhibitors may be a useful class of agent for targeting dormant cells thought to contribute to relapses in leukaemia.

## Abbreviations

7-AAD: 7-amino actinomycin D; AML: Acute myeloid leukaemia; araC: Cytarabine; CDK: Cyclin dependent kinase; DRB: 5,6-dicholoro-1-β-D-ribofuranoslybenzimidazole; EU: 5-ethynyl uridine; DCF: Dichlorofluorescein; DCFDA: 2′,7′-Dichlorofluorescin diacetate; FCS: Foetal calf serum; FSC: Forward scatter; HSC: Haematopoietic stem cell; mTOR: Mammalian target of rapamycin; rapa: Rapamycin; ROS: Reactive oxygen species; RP2: RNA Polymerase II; RP2S2: RNA Polymerase II serine 2; XTT: 2,3-bis(2-methoxy-4-nitro5-sulfophenyl)-5-{(phenylamino)carbonyl}-2*H*-tetrazolium hydroxide.

## Competing interests

Francis Burrows is an employee of Tragara Pharmaeuticals.

No financial interest/relationships with financial interest relating to the topic of this article have been declared by the remaining authors.

## Authors’ contributions

MP designed the study, participated in all experiments and drafted the manuscript. FB participated in the design of the study and contributed TG02. AW helped to develop the mTOR inhibition model. NB set up and participated in experiments to determine the effects of transcriptional CDK inhibitors on cell survival and RP2S2 phosphorylation. CS participated in the design of the study, oversaw the RNA experiments and participated in drafting the manuscript. NR participated in the design and co ordination of the study and contributed primary AML samples. All authors read and approved the final manuscript.

## Pre-publication history

The pre-publication history for this paper can be accessed here:

http://www.biomedcentral.com/2050-6511/14/32/prepub
